# The context of HIV risk behaviours among HIV-positive injection drug users in Viet Nam: Moving toward effective harm reduction

**DOI:** 10.1186/1471-2458-9-98

**Published:** 2009-04-06

**Authors:** Duong Cong Thanh, Karen Marie Moland, Knut Fylkesnes

**Affiliations:** 1 Centre for International Health, University of Bergen, Bergen, Norway; 2 Department of HIV/AIDS, National Institute of Hygiene and Epidemiology, Hanoi, Viet Nam; 3 Faculty of Health and Social Sciences, Bergen University College, Bergen, Norway

## Abstract

**Background:**

Injection drug users represent the largest proportion of all HIV reported cases in Viet Nam. This study aimed to explore the perceptions of risk and risk behaviours among HIV-positive injection drug users, and their experiences related to safe injection and safe sex practices.

**Methods:**

This study used multiple qualitative methods in data collection including in-depth interviews, focus group discussions and participant observation with HIV-positive injection drug users.

**Results:**

The informants described a change in the sharing practices among injection drug users towards more precautions and what was considered 'low risk sharing', like sharing among seroconcordant partners and borrowing rather than lending. However risky practices like re-use of injection equipment and 'syringe pulling' i.e. the use of left-over drugs in particular, were frequently described and observed. Needle and syringe distribution programmes were in place but carrying needles and syringes and particularly drugs could result in being arrested and fined. Fear of rejection and of loss of intimacy made disclosure difficult and was perceived as a major obstacle for condom use among recently diagnosed HIV infected individuals.

**Conclusion:**

HIV-positive injection drug users continue to practice HIV risk behaviours. The anti-drug law and the police crack-down policy appeared as critical factors hampering ongoing prevention efforts with needle and syringe distribution programmes in Viet Nam. Drastic policy measures are needed to reduce the very high HIV prevalence among injection drug users.

## Background

HIV transmission continues to be very high among injection drug users (IDUs) in many countries [1]. Studies among HIV-positive IDUs who know their HIV status show high rates of HIV risk behaviours including the sharing of needles and syringes [2-5], multiple sexual partners [4,6] and unprotected sexual intercourse [2-8]. A literature review based on publications from the US revealed that up to 64% of HIV-positive IDUs had shared injection equipment and 47% had engaged in unprotected sex during the previous 6 months [9]. Both injecting drug use and HIV among IDUs have spread to almost every country of the world, but only in a small number of countries have effective responses been implemented [10].

Being located in the outskirts of the Golden Triangle, Viet Nam has a long history of opium cultivation and smoking. Drug abuse expanded during the Viet Nam War (1959–1975) and became a big problem among US and South Vietnamese soldiers. However, it was not until the liberalization of the economy in the late 1980s and 1990s that the country faced serious problems associated with the production, trafficking and abuse of illicit drugs. The so-called 'renovation policy' stimulated economic growth, but also entailed increasing differences between rich and poor. Migration, commercial sex, drug abuse and traffic followed in its wake. Today there are about 135,000 IDUs in the country [10] and the prevalence of HIV among them is reported to be 29% nationally [11] but with wide geographical variations [12]. IDUs represent the largest proportion of all reported HIV cases in the country, accounting for 44% of the total [11].

In Viet Nam drug use is not only illegal, it is a "social evil", a perception deeply embedded in Viet Nam's culture and social norms [13,14]. The possession and sale of drugs are strictly illegal, and crackdowns, forced detoxification and imprisonment are common responses to violations of the law. Police and drug prevention and control programmes routinely raid the drug trafficking networks and the "shooting galleries" where drugs and injection equipment are sold or exchanged and where drug injection take place. Many are arrested and sent to drug rehabilitation centres ('06' centres) where they are subjected to detoxification, moral education to resist the drugs, health education on HIV and manual labour. In 2007 there were more than 50,000 residents in 06 centers in the country (about 25% of all drug users). The duration of stay varies from one to two years [13,14]. Commercial sex work is also illegal and female sex workers (FSWs) are met with similar reactions as IDUs. Unfortunately, these policies seem only to have minor effects on the magnitude of sex work. In a context where bridal virginity is highly valued men seem to continue having their first sexual experience with FSWs and many visit FSWs frequently [15,16].

Internationally a critical element of a public health response to HIV was harm reduction aiming to decrease drug-related harms. This accorded an even higher priority than reduction of drug consumption and involved explicit and peer-based education about the risk of HIV from sharing injecting equipment, needle syringe programmes, drug treatment including substitution treatment, and community development [17,18]. Viet Nam was slow to implement harm reduction interventions that targeted drug users. In the mid-1990s, with the support of international organizations, Viet Nam piloted several harm reduction measures including needle and syringe exchanges and methadone maintenance treatment. The national HIV strategy of 2004 supports harm reduction interventions for HIV prevention [13]. The National HIV Law of 2006 and Decree 108/2007 give directives for implementing harm reduction interventions including needle/syringe exchange, opioid substitution treatment and condom distribution. Implementation has been slow, however, owing to lack of sufficient political support and management capacity [13].

The present study was conduced in Hai Phong city, located in the economic developmental triangle of Quang Ninh-Hai Phong-Ha Noi, which is the centre of the HIV epidemic among IDUs in the north. The prevalence of HIV among IDUs in this area exploded in the late 1990s and seems to have peaked around 2001–2002, with 75% of IDUs being HIV positive in Quang Ninh in 2002, 72% in Hai Phong in 2001 and 31% in Ha Noi in 2004. Later observations indicated a decreasing trend, with HIV prevalence among IDUs of 54% in Quang Ninh, 46% in Hai Phong and 24% in Ha Noi in 2006. The HIV prevalence among antenatal women in Quang Ninh has exceeded 1% [11].

In response to the serious HIV epidemic among IDUs in the area, the local authorities in Hai Phong have developed intervention programmes e.g. peer education and self-help groups, and care and treatment programmes. The peer education programmes are outreach activities that include individual counselling, condom promotion, distribution of needles and syringes and distribution of educational pamphlets and brochures. The main purposes of the self-help groups are to reduce stigma and discrimination and to improve the quality of life of people living with HIV (PLHIV). The care and treatment programmes offer anti-retroviral therapy, treatment for opportunistic infections (OIs), and related services including medical provider training and capacity building for PLHIV.

In a previous survey among PLHIV in 20 provinces in Viet Nam we found clear indications of a high risk of HIV transmission from PLHIV to other IDUs, to female sex workers (FSWs) and to the general population owing to frequent sharing of injection equipment, frequent sexual mixing and sex work combined with low condom use [19]. In order to understand the context and the underlying mechanisms of the documented HIV risk behaviours better, we conducted a qualitative study among HIV-positive IDUs in Hai Phong using in-depth interviews, focus group discussions and participant observation. The objectives were to explore the perceptions of risk and risk behaviours among HIV-positive IDUs, and their experiences related to safe injection and safe sex practices.

## Methods

### Sampling methods

The study was conducted among HIV-positive IDUs in June and July 2007. The participants were selected from six districts in Hai Phong, including three urban areas (Hong bang, Ngo quyen and Le chan) and three sub-urban areas (An duong, An lao and Thuy nguyen). Local health workers and peer educators assisted in the recruitment of the study participants. Peer educators among the HIV-positive IDUs were identified through local health workers and PLHIV self-help groups, and were engaged to recruit the participants and to act as focus group discussion moderators. The peer educators were trained in the techniques of guiding focus group discussions and on how to approach possible study participants, what information to collect and how to collect it. To be included in the study, participants had to be at least 15 years old, to have injected drugs, to live in the study area and to be HIV positive.

### Data collection

Multiple qualitative data collection methods including in-depth interviews, focus groups and participant observation were used. These techniques allow for the systematic collection of information about sexual and drug injecting practices and about the settings for these practices. They enabled us to gain insights and in-depth information about the perceptions and experiences of HIV-positive IDUs. Moreover, the different techniques employed were complementary and served to cross-check each other, thus validating the findings. All focus group discussions and interviews were conducted in Vietnamese. The participants were given a small amount of money called "transportation support" as motivation and as a sign of appreciation.

The first author conducted in-depth interviews with fifteen participants. All interviews were conducted in a private place that facilitated private dialogue and that was not too noisy for tape recording. The in-depth interviews focused on individual perceptions and experiences.

Three focus group discussions were conducted in a private place convenient for the participants. The peer educators and the first author acted as moderators, facilitated the discussion and kept it focused on issues related to drug- and sex-related behaviours. Each focus group included 10 participants. Otherwise, efforts were made to balance the numbers of young and old, rich and poor, high and low education levels to secure a wide range of information. The focus group discussions focused on knowledge, common norms and values, perceptions and experiences and were used to clarify issues that had been raised in the in-depth interviews. All in-depth interviews and focus group discussions lasted for about one and a half hours. They were tape-recorded and then transcribed immediately after each session.

An interview guide for the in-depth interviews and a topic guide for the focus group discussions were developed. The main points discussed in both in-depth interviews and focus groups were: knowledge about the transmission of HIV and STDs and protection against them; types of drugs used frequently; injection practices; sharing behaviours; cleaning procedures; rationalizations of injection equipment-sharing behaviours; concern about transmission of HIV and STDs to sexual partners; use of protection methods; condom use with regular sex partners, FSWs and other casual sex partners; and reasons for not using condoms.

To complement the other two techniques, the first author undertook participant observation in places known as "shooting galleries" or "shooting areas" *(tụ điểm*). These included a park, a waste area, a public toilet and a railroad area. The observation of interaction and injection practices was important for the broader understanding of risk and behaviour, and of the challenges experienced by IDUs in reducing risk. The participant observation was done with guidance from a peer educator and a local health worker. The participants were approached and introduced by peer educators who also asked for permission to observe their injection practices. Furthermore their company was necessary to avoid police confrontation, particularly during crackdown campaigns.

### Study population

There were 45 study participants aged 21–45 years. The large majority were male; only five were female. Their educational level was fairly high; most had completed secondary school or higher (69%), and only two had not completed primary school. Unemployment was common; 18 (40%) had no work, 15 (33%) were workers, 6 (13%) were long-distance truck drivers, 3 (7%) were motorcyclists (providing transport services for local people), 2 (5%) were office staff, and 1 (2%) was a nurse. Of all participants, 19 (42%) were married, 17 (38%) were single, 5 (11%) were divorced and 3 (8%) were widowed. Only five (11%) participants were under anti-retroviral (ARV) treatment.

### Data management and analysis

Transcripts were read and reviewed multiple times by the first author who conducted the data analysis. The information was analysed and summarised according to predefined topic areas. Consensus on key issues (as well as non-consensus) was noted. For each study question, the most common and repeated answers and responses of the participants were summarised. Different points of view were also noted and considered. Discrepant ideas were carefully explored. Participants' similar examples and phrases were grouped for each theme. New and important themes were identified and added during the data analysis. The information obtained in the in-depth interviews and focus group discussions was analysed and merged according to topic areas. The work was then translated into English. The summarised data were reviewed by two authors to detect any important information that had been missed and to meet consensus of opinion. Illustrative quotations were then selected. The concept of "sharing" was defined as using needles and/or syringes previously used by other injecting users or lending them to someone else.

### Ethical issues

The study was conducted with the understanding and consent of each participant, and was approved by the Institutional Review Board of the National Institute of Hygiene and Epidemiology, Hanoi, Viet Nam. Participants were informed beforehand that the interviews would be tape-recorded and that the findings were confidential. No personal identifiers were collected.

## Results

In the following section we will describe how risk related to injecting drugs and sexual practices was perceived and handled, and how efforts to reduce risk were experienced among the study participants.

### Injecting drugs

The study participants perceived certain sharing practices as low risk while others were clearly considered high risk.

#### Low risk sharing – the rules of precautions

The informants consistently reported that sharing of syringes and needles was not frequent compared to what it had been in the past. They also observed a generation shift in injection practices. The young and relatively new drug users were less likely to report sharing and were more concerned about the risk of HIV transmission than the older and more experienced drug users. One of the more experienced drug users explained that:

*"Young people nowadays are more aware (tỉnh táo) than we were before" (a 32-year-old man, in-depth interview)*.

This awareness was seen to be connected not only to the public knowledge campaigns on HIV and drugs, but also to the experiences of HIV-positive IDUs whom they encountered in the shooting areas.

*"They (young or new IDUs) have learned from us a lot. Before, nobody was aware of HIV. Now, everybody is scared of getting it" (a 35-year-old man, focus group discussion)*.

Although sharing was reported to be less acceptable and less practiced than before, it was still known to be common among HIV-positive peers, as the following statement underscores:

*"Nowadays, most IDUs do not share except when they are both HIV positive" (a 34-year-old man, focus group discussion)*.

Sharing among HIV-seropositive concordant partners seemed to be commonplace and was hardly questioned at all as the following testimony demonstrates:

*"I share with my husband since we are both IDUs" (a 38-year-old woman, in-depth interview)*.

According to the informants the risk of becoming HIV infected and the risk of infecting others were concerns that guided their drug injection practices. HIV-positive IDUs seldom shared injection equipment with IDUs of negative or unknown HIV status. However, circumstances would at times force the drug addict to take risks. In such situations, where sharing was the unavoidable outcome, informants were concerned to protect others from HIV infection. This concern was clearly expressed in their distinction between borrowing and lending. In situations where no clean equipment was available, borrowing was more common than lending. Since they knew that they were HIV-positive, they only borrowed and seldom lent their syringes to others:

*"In an unavoidable situation, I borrow injection equipment from others and clean before use. But since I know that I already have HIV, I never lend syringes to others" (a 28-year-old man, in-depth interview)*.

Along the same line of thought, another informant explained who would inject first and last:

"I often inject drugs with some other IDUs. We are friends so I always let them inject first since I already have the disease" (a 31-year-old man, in-depth interview)

The precautions taken regarding with whom to share, the sequence of injection in the sharing (mixing) network according to HIV status, and the protection of others through borrowing rather than lending, were perceived as low-risk and responsible sharing behaviour that would limit the spread of HIV.

#### High risk sharing – 'syringe pulling'

Sharing syringes and needles without those precautions was considered high risk. It was observed that used syringes were often left after use at the shooting areas and that many IDUs collected left-over drugs from the used syringes to inject. When this issue was raised in the discussions and interviews, most participants did not care much since they believed that the ones who resorted to these risky practices would already be infected and were about to die. This so-called "syringe pulling" *(dồn xi)*, a slang word among IDUs, was regarded with abhorrence:

*"I threw my used syringes out on the railroad known as a shooting gallery. Some IDUs picked them up and collected the left-over drug to inject. I guessed those were the ones about to die" (a 31-year-old man, in-depth interview)*.

He added that in order to prevent this he usually bent the needles before throwing them away. Another informant added that people picked also up the used syringes for commercial reasons:

"I have seen a lot people in the railroad area picking up used syringes to collect left-over drug. They then sold the used syringes to the plastic collectors for recycling purposes." (a 30-year-old man, focus group discussion)

More personal accounts of desperate IDUs begging for the left-over drug also surfaced:

*"One day, there was one person helping me to inject. Then he asked me to have my used syringe to collect left-over drug. He also asked me do not pull blood (hồi)" (a 30-year-old man, focus group discussion)*.

Another situation that encouraged syringe pulling was hospitalisation, as the following example illustrates:

*"I never share. But when I was hospitalized in TB hospital I injected drugs. Some HIV-positive IDUs who were also hospitalized collected my used syringes. They only used water and pulled in and out several times to rinse" (a 30-year-old man, in-depth interview)*.

#### Barriers to harm reduction

These high-risk behaviours did not enjoy respect among the HIV-positive IDUs, but they were considered a result of structural barriers that limited the impact of the harm reduction interventions that had been put in place as part of the HIV prevention efforts in Hai Phong. Syringes and needles were distributed for free through peer educators. However, the study participants reported that they were reluctant to carry needles and syringes because this might get them arrested or even beaten up since they were used as evidence of illegal drug use:

*"If the police find you carrying needles and syringes they will take them away from you and even beat you" (a 29-year-old man, focus group discussion)*.

Many ended up buying the injection equipment in the shooting areas:

*"I receive needles and syringes from peer educators. But I do not want to carry them to the shooting areas since they may bring me trouble if I meet the police. Therefore, I usually buy them at the shooting areas" (a 31-year-old man, in-depth interview)*.

During focus group discussion and in-depth interviews, many of the peer educators reported that they had been denied access to the shooting areas where they were supposed to provide needles and syringes, counselling and support:

*"I am a peer educator and wear a uniform, but the police do not allow me to go to the shooting areas. They even beat me" (a 27-year-old man, focus group discussion)*.

Considerable distrust of police motives was reported. Money to buy drugs was termed "illegal money" and was likely to be confiscated in encounters with the police. As a young man recalled:

*"On that day, my friend and I had just got 300,000 VND (about 20 USD). We went to the railroad area to buy drugs. We encountered the police and got arrested. They took us to the police station and then took all of our money. They said this money is to buy drugs, so it is illegal money" (a 26-year-old man, focus group discussion)*.

The risk of getting arrested clearly represented a barrier to the distribution of free syringes and needles that was established in the area and motivated high-risk injection practices. A less controversial harm reduction intervention was the promotion of condoms. Although high-risk injection practices probably represented the most important threat to the efforts to contain the HIV epidemic in the area, sexual practices among HIV-positive IDUs constituted another challenge.

### Sexual practices

#### Reduced desire

The participants generally reported reduced sexual activity. Many had experienced a decrease in their sexual desires after they had been HIV infected for a period of time and some had experienced erectile dysfunction and ejaculation disorders. The use of female sex workers was obviously affected by this:

*"I do not have much sexual desire, but even so, my friends sometimes convince me to visit female sex workers" (a 32-year-old man, in-depth interview)*.

Hence, the use of female sex workers was limited:

*"Before, I went to visit female sex workers with my friends sometimes. But over the past two years, my sexual desires have gone" (a 31-year-old man, in-depth interview)*.

They also reported that they had no money to buy sexual services. They had to prioritise their scarce resources and the desire for drugs generally dominated over the desire for sex:

*"Most drug users often do not have money. If they have some money then they buy drugs. Drug desire is much stronger than sexual desire. So no drug user looks for female sex workers" (a 26-year-old man, focus group discussion)*.

However, informants also explained that buying sex was common among young people and even among HIV-positive IDUs who were healthy and who had a fair income to pay for the services as described in the quote below:

*"You know, men and youngsters, this kind of thing (having sex with female sex workers) cannot be missed, (laugh)" (a 31-year-old man, in-depth interview)*.

Hence, the use of female sex workers seemed to vary with the stage of the HIV disease, the severity of the drug problem and economic ability.

#### Condom use

Many participants said that they had not used condoms with their regular sex partners in the early stages after learning that they were HIV seropositive.

*"Before I disclosed my HIV status to my wife, I did not use condoms when we had sexual intercourse. I thought that she would already be infected since we had had unprotected sex many times before. In addition, it was difficult to tell her that I would use condom since we were just married" (a 30-year-old man, in-depth interview)*.

The fear of HIV disclosure was a major obstacle in condom use negotiations. Furthermore, informants reported that another difficulty was anticipated concordance of HIV status. As one woman, working as a street-based sex worker, explained:

*"I think I got HIV from my husband. Therefore, I do not use condoms with my husband even though he has not taken an HIV test yet" (a 32-year-old woman, in-depth interview)*.

In general, participants reported that condoms were not used among HIV concordant couples.

*"My wife and I are both HIV positive. So we do not use condoms" (a 31-year-old man, in-depth interview)*.

However, using condoms when having sex with female sex workers was expected:

*"Nowadays, female sex workers including street-based sex workers ask their clients to use condoms when having sex" (a 30-year-old man, focus group discussion)*.

Some of the informants said they always carried a condom in their wallet to be prepared. They did however distinguish between karaoke-based sex workers and street-based sex workers.

*"If you go to have sex with karaoke-based sex workers you have to use condom 100 percent" (a 38-year-old man, in-depth interview)*.

But condom use was also on the increase among street-based sex workers:

*"I always use a condom with my clients. I only do not use a condom when my clients ask not to use. I think he has already got the disease. But I prefer to use a condom since I may get other diseases when not using a condom. If I got the disease it will affect my job" (a 32-year-old IDU woman who works as a street-based sex worker, in-depth interview)*.

## Discussion

The present study was intended to explore the context and underlying mechanisms of HIV risk behaviours. Informants consistently reported that sharing of injection equipment among peers was less common now than in the past. However, injection equipment was reused and left-over drugs were used, and the heroin injection practice described as 'syringe pulling' or 'dồn xi' was a particular concern; it entails an extreme transmission risk. Needle and syringe distribution programmes were in place but were found to be seriously hampered by the anti-drug law and police "crack-down policy" since the IDUs were reluctant to carry needles and syringes to avoid being arrested. The informants who had been infected for a long time reported reduced sexual desire. Owing to lack of disclosure, low condom use during the early stages after individuals learned they were HIV seropositive placed their regular sexual partners at high risk of HIV infection. Frequent borrowing of injection equipment among peers and low condom use with HIV concordant partners suggests a high risk of HIV superinfection and infection with other blood-borne diseases in this particular group.

The observation that sharing of injection equipment had decreased over time agrees with previous findings from Viet Nam [20-22] and is consistent with an observed decline in HIV prevalence among IDUs in recent years both as a national trend and in Hai Phong. This is encouraging and may to some extent be a result of intervention efforts, but needle sharing and the prevalence of HIV among IDUs [22] is still extremely high indicating that the prevention programmes are not effective.

In the literature, sharing is often defined as using needles and/or syringes previously used by other injecting users or lending them to someone else. 'Syringe pulling' should also be considered as indirect sharing of injection equipment and involves a risk of spreading beyond peers. Furthermore, improper injecting equipment disposal may be harmful to the environment and the community at large. Local residents and plastic collectors for recycling purpose may be exposed to HIV and other blood-borne diseases through needle stick-related accidents. Therefore, continuing behavioural intervention strategies and policies addressing drug use-related harm reduction are urgently needed, e.g. safe injecting practices and safe disposal of used needles and syringes.

As in previous studies [20,23], our data suggest that the anti-drug law and police crack-down policy constitute an important barrier to clean needle and syringe distribution programmes. IDUs were reluctant to carry needles and syringes to avoid this owing to fear of being arrested and fined, although the needles and syringes were given by peer educators. Accordingly, IDUs mostly relied on drug dealers for their supply of drugs and injection equipment and were therefore forced to inject right away. There is no evidence that shortages of injection equipment will diminish the use of injected drugs [24]. If there are shortages of injection equipment among drug dealers then sharing is likely to increase. Also, a previous study showed that a non-supportive environment for safer practices contributes to continued needle sharing [20]. Previous research has shown that the needle and syringe distribution programme was not successful in reducing sharing behaviour in Viet Nam [25]. There is a need for multi-sectoral collaboration and communication among ministries and agencies involved in HIV and drugs programmes to create policies for supportive environments for changes in both drug use and HIV risk behaviours.

Fear of rejection and of loss of intimacy resulted in difficulty in disclosing serostatus to sexual partners and finally to putting those partners at risk. This observation is similar to previous findings showing that nondisclosure is associated with increased sexual risk practices [26,27]. The present study found a decrease in sexual desire among those who had been HIV infected for a long time, but our previous survey indicated that many PLHIV were still sexually active and used condoms inconsistently [19]. Increased sexual activity due to improved health status after ARV therapy has been observed, and perceptions of reduced infectivity are associated with increased HIV sexual risk behaviour [28,29]. Voluntary HIV counseling and testing has been shown to be an effective HIV prevention strategy and the entry point to care and support for PLHIV [30-33]. In our survey among PLHIV we found that those who received voluntary HIV testing were more likely to report reduced risk behaviour [19]. The need for HIV risk reduction counselling for PLHIV, therefore, should be emphasized.

Our data suggest that the study participants did not seem to recognize or even understand the risks of HIV superinfection and recombination [34-37]. HIV-positive IDUs were willing to have sex without a condom and to share injection equipment with HIV concordant partners. Unprotected sex and sharing of injection equipment among HIV-positive IDUs thus place them at risk of being infected with new HIV strains and other blood-borne diseases that could increase the progression of the disease. For instance, chronic hepatitis C has been found to have a worse prognosis in HIV-infected patients because of the higher risk and more rapid progression of cirrhosis, a higher risk of end-stage liver decompensation, and liver-related death [38]. More research is needed to clarify the risk of HIV super infection, recombination and coinfection, e.g. hepatitis B and hepatitis C. Furthermore, HIV education programmes should stress the importance of safer behavioural practices regardless of one's partner's serostatus.

## Conclusion

The study findings suggest that HIV-positive IDUs continued to practice HIV risk behaviours that put others at risk for HIV infection and themselves at risk of HIV superinfection and recombination as well as blood-borne coinfections. The 'social evil" approach and illegality of drug use have driven IDUs into a very marginalized situation. The anti-drug law and the police crack-down policy critically hamper ongoing prevention efforts with the needle and syringe distribution programme in Viet Nam. Although there are clear signs of decrease in HIV prevalence and needle and syringe sharing, drastic policy changes are needed to reduce the very high HIV prevalence among IDUs. Currently, the HIV epidemic in Viet Nam is still concentrated primarily among IDUs and FSWs [23]. Therefore, this opportunity for effective intervention to prevent a generalized epidemic should not be missed.

## Competing interests

The authors declare that they have no competing interests.

## Authors' contributions

All authors contributed to the paper, all authors conceived the study. DCT conducted the study. All authors helped to conceptualise ideas and interpret the findings. DCT prepared the drafts, and other authors reviewed and finalise the manuscript.

## Pre-publication history

The pre-publication history for this paper can be accessed here:


